# The serotonin transporter gene and female personality variation in a free-living passerine

**DOI:** 10.1038/s41598-021-88225-4

**Published:** 2021-04-21

**Authors:** Bert Thys, Andrea S. Grunst, Nicky Staes, Rianne Pinxten, Marcel Eens, Melissa L. Grunst

**Affiliations:** 1 Department of Biology, Behavioural Ecology and Ecophysiology Group, University of Antwerp, Campus Drie Eiken, D.D. 123, Universiteitsplein 1, 2610 Wilrijk, Belgium; 2 Littoral Environnement et Sociétés, La Rochelle Université, La Rochelle, France; 3 Centre for Research and Conservation, Royal Zoological Society of Antwerp, Antwerp, Belgium; 4 Faculty of Social Sciences, Antwerp School of Education, University of Antwerp, Antwerp, Belgium

**Keywords:** Behavioural genetics, Animal behaviour, Behavioural ecology, Ageing

## Abstract

Quantifying variation in behaviour-related genes provides insight into the evolutionary potential of repeatable among-individual variation in behaviour (i.e. personality). Yet, individuals typically also plastically adjust their behaviour in response to environmental conditions and/or age, thereby complicating the detection of genotype–phenotype associations. Here, using a population of free-living great tits (*Parus major*), we assessed the association between single nucleotide polymorphisms (SNPs) in the serotonin transporter gene (*SERT*) and two repeatable behavioural traits, i.e. female-female aggression and female hissing behaviour. For female-female aggression, a trait showing age-related plasticity, we found no evidence for associations with *SERT* SNPs, even when assessing potential age-dependent effects of *SERT* genotype on aggression. We also found no strong support for associations between *SERT* SNPs and hissing behaviour, yet we identified two synonymous polymorphisms (exon 13 SNP66 and exon 12 SNP144) of particular interest, each explaining about 1.3% of the total variation in hissing behaviour. Overall, our results contribute to the general understanding of the biological underpinning of complex behavioural traits and will facilitate further (meta-analytic) research on behaviour-related genes. Moreover, we emphasize that future molecular genetic studies should consider age-dependent genotype–phenotype associations for behavioural trait (co)variation, as this will vastly improve our understanding of the proximate causes and ultimate consequences of personality variation in natural populations.

## Introduction

Individuals within single populations are often found to vary in their average behaviour across repeated observations, known as animal personality^1,2^. Traditionally, this among-individual behavioural variation was often attributed to random (i.e. non-adaptive) noise around an adaptive mean^3^. Today, it is apparent that personality can be heritable^4–6^, influence fitness^7,8^ and therefore potentially evolve adaptively in response to selection^9,10^. Although quantitative genetic studies have revealed substantial genetic variation underlying variation in personality traits (e.g.^4,5^), our knowledge about the specific genetic architecture of personality variation still remains in its infancy^11–13^. Yet, understanding how genes contribute to the shaping of behavioural phenotypes is essential to assess their evolutionary potential and how personality variation is maintained in natural populations.

Besides being repeatable, behavioural trait expression is typically also plastic, with individuals adjusting their behaviour in response to external (e.g. environmental) and internal (e.g. age) factors^14,15^. Consequently, effects of genes on behavioural phenotypes (and hence heritability) might not be constant across environments and/or the life span of individuals, as characterized by genotype-by-environment (i.e. G × E) and genotype-by-age (i.e. G × A) interactions, respectively^15,16^. In other words, effects of certain genes or gene polymorphisms on behavioural phenotypes might be reduced, shut down or altered under specific environmental conditions and/or at certain ages, indicating that genes can act in an environment- and/or age-dependent manner (e.g.^17,18^). Although still limited in number, studies in natural populations are increasingly revealing heterogeneity in the associations between genetic polymorphisms and behavioural traits across populations, suggesting the existence of G × E in behaviour (e.g.^19–21^; see also^22,23^). In contrast, studies investigating G × A in behaviour, which can be considered a specific form of G × E, are very scarce (^24,25^; review in^15^), and we are unaware of any study assessing whether the association between specific genes (or polymorphisms) and behavioural traits interact with age in natural populations. Modelling the interaction between genotype and age is important for traits that show age-related phenotypic plasticity since the latter can complicate the detection of associations between genes and behavioural phenotype.

A variety of so-called candidate genes have been hypothesized to be involved in the expression of particular behavioural phenotypes (recently reviewed in^13^). One promising candidate gene particularly with regard to anxiety- and aggression-related behaviour is the serotonin transporter gene (*SERT*) (e.g.^4,12,13,26,27^). The serotonin transporter has an important role in the regulation of extracellular and synaptic serotonin concentrations, and hence the magnitude and duration of serotonergic neurotransmission. In human and non-human primates, allelic variation in *SERT* has been associated with anxiety, harm avoidance, dominance, aggression and sexual behaviour^26–29^. Additionally, a handful of recent studies suggest that single nucleotide polymorphisms (SNPs) in *SERT* can be associated with behavioural variation in free-living bird species. Notably, SNPs in *SERT* have been associated with variation in flight initiation distance in dunnocks (*Prunella modularis*^23^) and novel object and anti-predator responses in great tits (*Parus major*^21,30,31^; but see also^32,33^).

Here, using a population of free-living great tits, we aimed to identify genomic variation underlying two female personality traits, that is, female-female aggression and female anti-predatory nest defence (so-called hissing behaviour^34^). In this population, both behavioural traits have been shown to be short-term (i.e. within-year) and long-term (cross-year) repeatable, but to not covary on the among-individual level (i.e. no behavioural syndrome^35–37^). The latter raises the, as of yet untested, possibility that aggression and hissing behaviour are influenced by different underlying genetic mechanisms. Moreover, we recently revealed that female-female aggression, but not hissing behaviour, decreased within individuals with age and that individuals differed in their level of age-related plasticity in aggression (i.e. individual-by-age interaction; I × A;^37^). Since G × A can underlie I × A^15^, the influence of genes on aggression might vary with age, but whether this is the case remains to be evaluated. Hence, using a behavioural dataset collected over four years (see^37^) and *SERT* as a candidate gene, we assessed the association between *SERT* SNPs and both aggression and hissing behaviour in female great tits. For aggression, we additionally assessed whether the potential associations with SNPs interacted with age, which would be characterized by age-dependent aggression-SNP associations (i.e. G × A).

## Methods

### Study population and standard procedures

Data were collected in a semi-urban population of free-living great tits in the surroundings of Antwerp, Belgium (Fort 6/Campus Drie Eiken; 51° 09′ 44″ N–4° 24′ 15″ E), which has been monitored since 1997 and at present consists of approximately 150 nest boxes for great tits. As part of long-term monitoring, all birds in the population are provided with a metal leg ring as nestlings or upon first capture (in winter or when feeding nestlings), and all adults receive a unique combination of three plastic colour rings. A blood sample (20 µl) is collected from the brachial vein for all birds, either as nestling or as adult upon first capture. Reproductive activities of all breeding pairs are monitored throughout the nesting cycle to determine lay date, clutch size and onset of incubation.

### Behavioural measurements

For four years (2016–2019), behavioural experiments were performed on females with first clutches^37^. First, female-female aggression was assessed using simulated same-sex territorial intrusion tests (henceforth aggression test), following^35^. In short, a stuffed female great tit (decoy, one of five) was placed on top of the focal female’s nest box at day 2 and 5 of the egg-laying period (with day 1 the day the first egg was laid). The focal female’s behaviour was observed for 5 min, starting when she entered within a 15 m radius around the nest box, or when she was already present at the start of the test. The observer (B.T. or an observer trained by B.T., 11 in total), standing at a distance of approximately 15 m, scored the following aggression parameters using a customized handheld tally counter device: the number of alarm calls produced, the time spent on the decoy (in s) and the number of attacks towards the decoy. Also, approach distance (in m) was scored, representing the minimum distance of the focal female to the decoy during the observation period.

Second, when confronted with a predator inside the nest cavity, some incubating and brooding females produce loud broadband hissing calls, often accompanied with intense flapping of the wings and lunging at the predator^34^. This so-called hissing behaviour was assessed using simulated predator intrusion tests (henceforth hissing test), following^38^. Specifically, the observer (one of 11) entered the head of a stuffed great spotted woodpecker (*Dendrocopos major*; one of three) into the entrance hole of a focal female’s nest box, at day 2 and 5 of the incubation period, thereby blocking the only entrance to the nest box and preventing the incubating female from escaping. The woodpecker was held in this position for one minute, during which the number of hissing calls produced were counted, which can easily be heard from outside the nest box. The number of hissing calls produced was used as a measure of hissing behaviour.

Over the course of 2016–2019, a total of 686 aggression tests and 866 hissing tests were successfully performed on 290 and 311 unique females, respectively, with 289 of these females tested for both behaviours during the same breeding attempt (details on data structure can be found in Table 1 in^37^). Age of females (with age = 0 representing age of birth) was determined using hatching records (resident birds) or plumage characteristics upon first capture (first-year or older). Absolute age was therefore known for all local recruits (N = 96 out 312 females; 30.8%) and immigrant birds first captured as first-year-olds (N = 192; 61.5%). For immigrant birds with an adult plumage upon first capture (N = 24; 7.7%), we assumed they recruited into the population as 2-year-olds^37^. We were able to genotype 306 of the 312 females in our behavioural dataset (*detailed below*).

### Genotyping

DNA was extracted from 306 blood samples using a commercial kit (Macherery-Nagel NucleoSpin® blood kit), following manufacturer’s instructions. Next, using polymerase chain reactions (PCRs), we amplified the 13 exonic regions of *SERT*. New primers were designed to optimize reaction performance for exon 1 and 12 using Geneious Prime 2020.0.4, but otherwise primers were derived from^30^ (see Supplementary Table S1). Thermocycling condition for exons 2 through 13 were 95 °C for 15 min, followed by 38 cycles of 95 °C for 30 s, 52 °C for 30 s, and 72° for 1 min, with a final extension at 72 °C for 4 min. Since exon 1 has longer product length, the themocycling conditions were 95 °C for 15 min, followed by 38 cycles of 95 °C for 30 s, 54 °C for 30 s, and 72° for 1 min, with a final extension at 72 °C for 6 min. PCR reactions were run on a Mastercycler gradient PCR machine (Eppendorf) for all exons, expect for exon 2. The reaction mixture (15 µl) consisted of 7.5 µl of master mix (HotStarTaq Master Mix, Qiagen), 0.6 µl of both the forward and reverse primers at a concentration of 10 µM, 3.3 µM of DNase free water, and 3.0 µl of DNA at a concentration of 10 µM. Exon 2 was amplified on a LightCycler480 (Roche), using a reaction mixture (15 µl) consisting of 7.5 µl of master mix (LightCycler480 High Resolution Melting Master), 0.525 µl of the forward and reverse primer at 10 µM, 1.2 µl of MgCl_2_ (concentration in master mix, 2 µM), and 2.0 µl of DNA at 10 µM.

Genotypes were determined via direct sequencing of PCR amplicons, performed at the genomics core facility of the University of Antwerp using a sanger sequencing platform. Sequences where aligned to the great tit reference genome using Geneious Prime 2020.0.4 and single nucleotide polymorphisms (SNPs) were identified by manual inspection. At first, a subsample of 46–48 females (selected based on large variation in the focal behavioural traits) were genotyped across all loci, and sequenced in both directions (i.e. forward and reverse primers). This revealed SNPs with genotype frequencies > 5% in 8 exonic regions, i.e. exons 1, 2, 3, 5, 6, 9, 12 and 13. All other exons either contained rare SNPs (< 5%; exons 7, 8, 10 and 11) or were monomorphic (exon 4), and were not further considered. Next, all other females in the dataset were genotyped for the eight polymorphic exons, which were sequenced in the direction that gave the best coverage across a particular locus (i.e. either forward or reverse). Sample sizes differ somewhat between loci due to sequencing failures of some SNPs for some individuals.

### Genotypic quality control

Within the eight exonic regions, we excluded SNPs in which minor allele frequencies (MAF) were < 10% (Supplementary Table S2), to avoid small numbers of individuals in any genotype and/or genotype-age class. All remaining SNPs were tested for deviation from Hardy–Weinberg equilibrium (HWE) using chi-square tests (*Hardy–Weinberg* package in R^39^). Linkage disequilibrium (LD) statistics (D’ and r) among the remaining SNPs within *SERT* were estimated using SNPStats^40^ (Supplementary Table S3). Significance levels for HWE and LD statistics were adjusted for false discovery rate (FDR; *p.adjust* function in R^41^).

### Statistical analyses

First, a principal component analysis was performed on the aggression parameters (i.e. number of alarm calls produced, approach distance, attacks and time on decoy) scored during territorial intrusion tests^35^. This resulted in a single principal component (PC1) with eigenvalue > 1 (EV = 1.40), explaining 49% of variance. High scores on PC1 correspond to closer approach distance, more time on the decoy and more attacks. In contrast, low scores on PC1 correspond to the production of more alarm calls from a larger distance (Supplementary Table S4). Scores on PC1 were used as a measure of aggression in all further analyses (henceforth aggression).

Second, association testing between each *SERT* SNP and behavioural traits was performed using linear mixed models fitted with the *lmekin* function (*coxme* package in R^42^). For each behavioural trait, we fitted two standard allele effect models, i.e. the additive and the overdominant effect model, thereby covering the complete range of allele effects (additive, recessive, dominant and overdominant^20,43^). Genotype was coded as a continuous covariate (three-levels; 0, 1 or 2 copies of the minor allele) in the additive effect models, and as a two-level factor (homozygote = 0; heterozygote = 1) in the overdominant effect models. Based on previous findings, we also included lay date (relative to the first-egg date in the given year) and clutch size as fixed effects in the models for hissing behaviour^36,37^. Clutch size was centred and standardized within individuals, thereby partitioning effects of clutch size on behavioural traits into its among-individual (i.e. mean clutch size per individual) and within-individual (clutch deviation; i.e. the deviation of each observation from an individual’s mean clutch size) components^44^. For aggression, we included age and its two-way interaction with genotype as fixed effects. We pooled individuals of 3 years old or older into one age-class to avoid small numbers of individuals in any genotype-age class (i.e. three age-classes: 1, 2 and 3+). Nonetheless, for certain SNPs this still resulted in a small number of individuals in certain genotype-age classes and the interaction between age and genotype was only included in the models where sufficient individuals (≥ 5) were available in each genotype-age class (see Supplementary Table S5). Year (2016–2019) was included as a fixed effect in all models to control for annual variation^37^. Other fixed effects previously shown to not affect variation in behavioural traits (e.g. time and date of behavioural testing) were not included^35,36^. All models included random intercepts for female identity (ID) and the unique combination of ID and year (ID_Year), the latter denoting a period (i.e. breeding season) during which repeated observations were obtained for individuals (see^37,45^). To control for the effect of relatedness among females, we constructed a relatedness matrix (*kinship* function, *kinship2* package in R^46^) using pedigree information based on social matings (2004–2019) and entered this matrix into all models. Relatedness was modelled this way because, due to high immigration and dispersal, we failed to collect behavioural data for a sufficient number of related females to fit animal models (i.e. the pedigree was not well-connected; see^47^). We also did not include random intercepts for observer or decoy identity since previous results using the same behavioural dataset revealed that they explain little to no variation in focal behavioural traits (see^37^). For significant SNPs we calculated the proportion of variance in behaviour explained using the marginal coefficient of determination (R^2^_m_^48^).

All analyses were performed in R 3.6.1 (R core team, 2019). Response variables were standardized to unit variance prior to analyses and, based on visual inspection of model residuals, all models were fitted assuming Gaussian error distribution. Interaction effects were removed from final models when non-significant. Results are presented as means with standard error (SE), unless stated otherwise, and we report significance levels of effects before (P-value) and after adjustment for FDR (P.adjust).

### Ethical statement

This study was approved by the ethical committee of the University of Antwerp (ID 2017-23 and 2017-61), performed in accordance with Belgian and Flemish laws regarding animal welfare, adhered to the ASAB/ABS guidelines for the use of animals in behavioural research and teaching, and complies with ARRIVE guidelines. The Royal Belgian Institute of Natural Sciences (KBIN) provided ringing licences for all authors and technicians.

## Results

### Genetic polymorphisms

We detected *SERT* SNPs at 27 loci across 8 exonic regions in our population (Supplementary Table S2). For 15 of these SNPs minor allele frequency was < 10% and an additional 4 SNPs deviated significantly from HWE even after correcting for FDR, so they were removed from further analyses. Hence, we retained 8 SNPs across 5 exons for association testing with behavioural traits (Table 1). Of these 8 SNPs, SNP226 in exon 1 is non-synonymous (i.e. causing changes in amino acids), while all other SNPs are synonymous (Table 1). We found no complete linkage disequilibrium between SNPs. Yet, strong linkage disequilibrium (P.adjust < 0.01) was found among the three SNPs within exon 1, between the two SNPs within exon 9, and among the two SNPs in exon 9 and the SNP in exon 13. For all other pairs of SNPs linkage disequilibrium was weak (see Supplementary Table S3 for full results).

**Table 1:** Single nucleotide polymorphisms (SNPs) in *SERT* with minor allele frequency (%m) > 10%.

Locus	Coordinate	Location	N	M/m	mm	Mm	MM	%m	χ^2^	P.adjust	Protein coding	AA change
SNP106	chr19:5978897	Exon 1	286	G/A	12	61	213	14.86	6.02	0.04	Non-coding	
**SNP163**	chr19:5978840	Exon 1	286	G/A	5	57	224	11.71	0.13	0.79	Synonymous	
**SNP187**	chr19:5978816	Exon 1	289	C/T	11	98	180	20.76	0.14	0.79	Synonymous	
**SNP226**	chr19:5978777	Exon 1	289	T/A	32	114	143	30.80	1.34	0.50	Non-synonymous	E26D
SNP101	chr19:5976872	Exon 3	303	A/G	18	30	255	10.89	68.24	< 0.001	Synonymous	
SNP125	chr19:5976812	Exon 3	301	A/G	17	45	239	13.12	30.57	< 0.001	Synonymous	
SNP187	chr19:5976777	Exon 3	303	A/T	17	50	236	13.86	29.70	< 0.001	Non-synonymous	L260Q
**SNP36**	chr19:5973968	Exon 6	286	T/C	72	140	74	49.65	0.07	0.79	Synonymous	
**SNP51**	chr19:5971865	Exon 9	284	C/T	15	88	181	20.77	0.70	0.60	Synonymous	
**SNP84**	chr19:5971832	Exon 9	289	T/C	58	124	107	41.52	3.57	0.14	Synonymous	
**SNP144**	chr19:5968682	Exon12	303	C/T	6	57	240	11.39	0.86	0.60	Synonymous	
**SNP66**	chr19:5967914	Exon13	302	C/T	26	115	161	27.65	0.52	0.63	Synonymous	

Coordinate refers to the position within the great tit genome on chromosome 19. For each SNP we give the total sample size (N), major/minor alleles (M/m) with sample sizes per genotype and protein coding with associated amino acid (AA) changes. Chi-square (χ^2^) statistics for Hardy–Weinberg equilibrium are given with associated significance levels adjusted for false discovery rate (P.adjust). SNPs used for association testing with behavioural traits are depicted in bold.

### Associations between SNPs and behaviour

**Figure 1:**
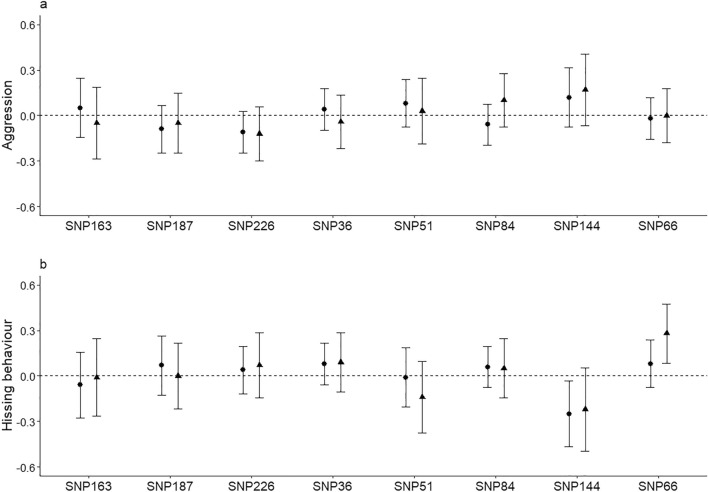
Effect size with 95% confidence intervals (CIs) of *SERT* SNPs for female-female aggression (**a**) and hissing behaviour (**b**) from the additive effect (circles) and the overdominant effect (triangles) models. For the additive model, positive effects indicate the average increase, and negative effects the average decrease in aggression/hissing behaviour with successive replacement of alleles. For the overdominant model, positive effects indicate the average increase, and negative effects the average decrease in aggression/hissing behaviour from homozygous individuals to heterozygous individuals. Effects are significant (with P < 0.05, but P.adjust > 0.05) when CIs do not overlap with zero (dotted lines).

No evidence was found for significant associations between aggression and *SERT* SNPs in either the additive or overdominant effect models (Fig. 1a). Additionally, although aggression decreased across age-classes, we found no support for effects of the interaction between genotype and age-class on aggression (Supplementary Table S5).

**Figure 2:**
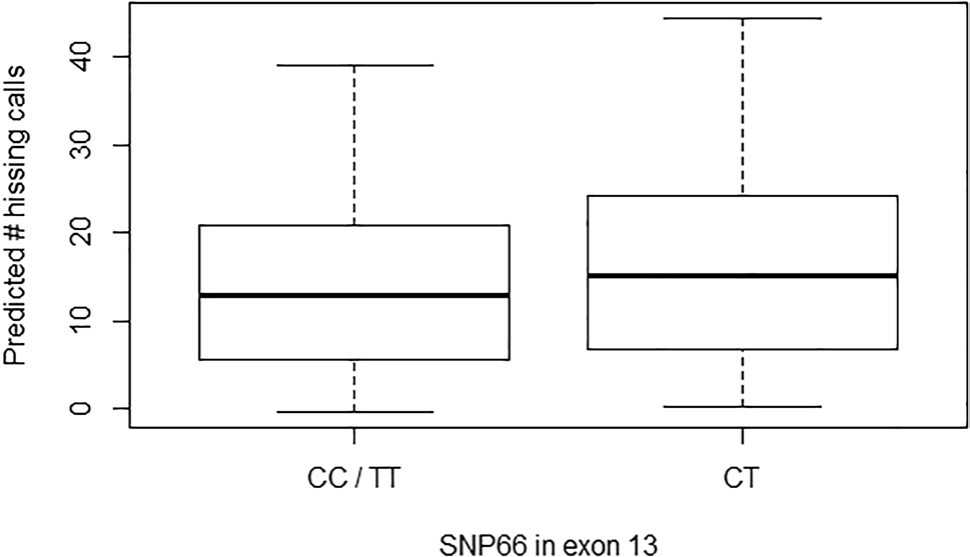
Quartile based box plots of the number of hissing calls produced by females in relation to exon 13 SNP66 genotype. Plotted are the number of hissing calls corrected for fixed and random effects included in the overdominant effect model (i.e. predicted values; see text for details). For illustration purposes, the estimates from the model with standardized hissing calls were back-transformed to the actual number of hissing calls produced. N females per genotype: CC/TT: 186, CT: 113.

**Figure 3:**
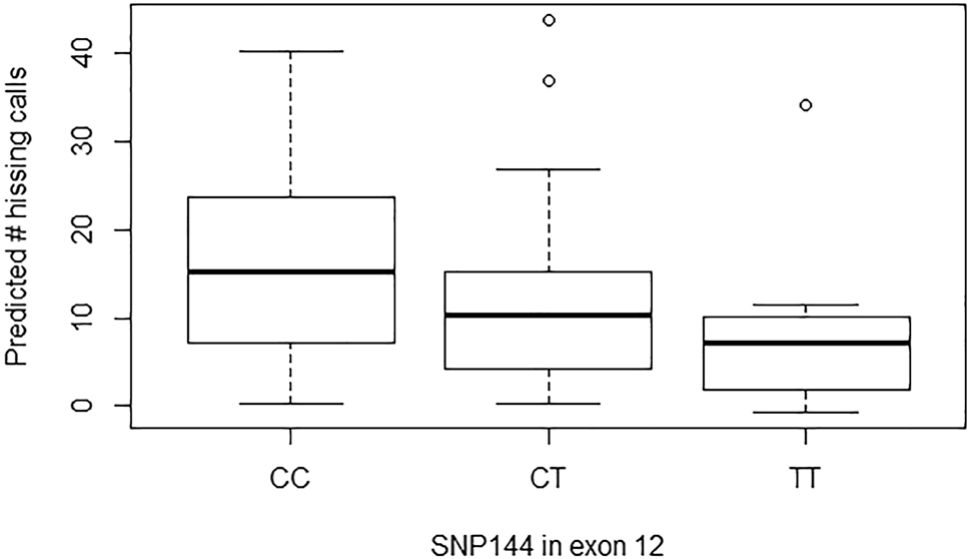
Quartile based box plots of the number of hissing calls produced by females in relation to exon 12 SNP144 genotype. Plotted are the number of hissing calls corrected for fixed and random effects included in the additive effect model (i.e. predicted values; see text for details). For illustration purposes, the estimates from the model with standardized hissing calls were back-transformed to the actual number of hissing calls produced. N females per genotype: CC: 238, CT: 56, TT: 6.

Overall, we also found no strong support for associations between hissing behaviour and *SERT* SNPs (Fig. 1b; Supplementary Table S6). Notably though, there were two hissing behaviour—*SERT* SNP associations that deserve particular attention. First, the association between variation in hissing behaviour and exon 13 SNP66 genotype was significant before, and marginally non-significant after, controlling for FRD in the overdominant effect model (0.28 ± 0.10; P = 0.007; P.adjust = 0.054), where SNP66 genotype explained 1.3% of the total variance in hissing behaviour (R^2^_m_ = 0.013). Specifically, homozygous females for SNP66 produced on average 3.26 hissing calls less compared to heterozygous females (Fig. 2). Second, the association between variation in hissing behaviour and exon 12 SNP144 genotype was significant before, but not after, controlling for FDR in the additive effect model (β ± SE: − 0.25 ± 0.11; P = 0.023; P.adjust = 0.184; R^2^_m_ SNP144 = 0.013). Specifically, the number of hissing calls produced decreased on average by 2.92 with the successive replacement of C-alleles in SNP144 (i.e. CC → CT → TT; Fig. 3).

## Discussion

Identifying genes that underlie personality can improve our understanding of how among-individual variation in behaviour is maintained in natural populations. Here, in a population of free-living great tits, we found no evidence for associations between *SERT* polymorphisms and female same-sex aggression, even when potential age-dependent effects of *SERT* genotype on aggression were assessed (i.e. no evidence for genotype-by-age interaction; G × A). In general, we also found no strong support for associations between *SERT* polymorphisms and hissing behaviour. Yet, genomic sequence variation at one synonymous locus in *SERT* (SNP66 in exon 13) showed a strong, marginally non-significant, tendency to be associated with variation in hissing behaviour. Another synonymous polymorphism (SNP144 in exon 12) also appeared to be associated with hissing behaviour, although not significantly so after correcting for multiple testing. As expected for single loci influencing complex behavioural traits, each of these SNPs explained a small amount (1.3%) of the total variance in hissing behaviour. Overall, our results therefore suggest that the SERT gene might be involved in heritable variation in hissing behaviour, but not in female-female aggression, although caution is warranted given the possibility for false positive results (i.e. type I errors).

Although we have previously demonstrated that female-female aggression is both repeatable and plasticity adjusted according to age^37^, we found no evidence for (age-dependent) associations with genomic sequence variation in the exonic regions of *SERT*. It should be noted that for some SNPs there were very few females in certain genotype-age classes and whether genotype-by-age interactions occur for these SNPs remains unknown. Moreover, we cannot rule out that factors other than age, such as external environmental conditions, may result in induced or inherited changes in a gene’s expression^49^. For example, in free-living great tits, dopamine receptor D4 (*DRD4*) genotype was associated with exploratory behaviour in only one out of four populations, potentially explained by cross-population environmental differences modifying genetic effects^19^. More recently, environmentally induced epigenetic modifications (i.e. DNA methylation) have been suggested to play a role in the association between *SERT* genotype and novelty responses across urban and rural great tit populations^21^. Hence, it will be interesting to investigate whether genotype-by-environment interactions occur for the association between female same-sex aggression and *SERT* polymorphisms, both within and across populations.

Overall, we also found no strong support for associations between hissing behaviour and *SERT* polymorphisms. Yet, variation in hissing behaviour was marginally non-significantly associated with genomic sequence variation in exon 13 (SNP66), where homozygous females produced on average less hissing calls compared to heterozygous females. Additionally, we revealed some tentative indications for an additive genetic effect of SNP144 in exon 12 on hissing behaviour. As noted before, these associations might represent false positive results (i.e. type I errors), especially in the case of exon 12 SNP144 as the latter association was far from reaching significance after correcting for multiple testing. Nonetheless, our results are informative for studies aiming to reconcile the effects of *SERT* on behaviour, including those aiming to identify biological pathways involved in the expression of hissing behaviour. That is, the here identified SNPs in exon 12 and 13 were not detected in another great tit population where the association between hissing response and *SERT* SNPs was assessed^31^. Instead, the latter study found that *SERT* SNP187 in exon 1 explained about 16% of the variation in whether or not (i.e. binary response) females produced hissing calls upon predator confrontation. Although we found a SNP at the same location in exon 1 (SNP187), this was not associated with the same nucleotide change (i.e. C/T instead of A/T; Table 1) and not with variation in hissing behaviour. Different results across these two populations may be due to cross-study differences in the quantification of hissing responses (i.e. binary versus continuous response) and/or a combination of type I and type II errors (^31^; but see^11,50^). Nonetheless, genuine cross-population differences in gene-behaviour associations are common^12,13,26,50^ and can be the result of, amongst other factors, divergent selective pressures, different mutations, and gene-by-environment interactions^19,20,51^. Notably, since the proportion of females not producing hissing calls (across all repeated observation) can greatly differ among great tit populations (e.g. our population: 7.7%; Estonian population: 48%^31^; Latvian population: 30%^52^), it will be of interest to investigate whether population differences in *SERT*-hissing behaviour associations represent genuine population-specific effects, preferably across a large number of populations.

As expected for quantitative behavioural traits, single loci had only small effects on hissing behaviour in our population, each explaining about 1–2% of variation^11,12^. Although small from a statistical viewpoint, from a genetic point of view these effects can be non-negligible and substantial, particularly for a genetic association with complex behavioural traits^19,53^. Hence, the here identified SNPs with small effects can guide our further understanding of the biological underpinning of hissing behaviour^12,13,26^. At present it indeed remains unclear how these potential effects come about, especially since the associated polymorphisms are synonymous and do not determine amino acid sequences of the encoded protein. However, there is growing evidence that synonymous polymorphisms can have functional effects via their influence on transcription, splicing, mRNA stability or translation, any of which could alter the phenotype^54,55^. Alternatively, synonymous SNPs can be linked with variation in non-coding regions that influence a gene’s expression, or with functionally significant polymorphisms (i.e. non-synonymous SNPs) in other genes^54,56^. Hence, a next step would be to investigate linkage disequilibrium patterns between exonic SNPs (especially SNP66 in exon 13) and variation in intronic regions of *SERT*, as well as in the wider gene region (i.e. flanking regions and adjacent genes; see e.g. ^20^).

In general, our study identified SNPs in *SERT* that might be involved in the expression of hissing behaviour, but not female same-sex aggression. Different findings for different traits might be related to the behavioural context in which they are expressed. That is, growing evidence in rodents, humans and non-human primates suggests that different forms of aggression could be influenced by different components of the serotonergic system, as well as interactions with other neurochemical systems^27,57–59^. In the case of female-female aggression, females can decide whether or not to (aggressively) engage during same-sex conspecific confrontation in their territory, therefore likely reflecting a female’s tendency for offensive aggression. In contrast, hissing behaviour is typically expressed in situations where predator confrontation is unavoidable due to predators often blocking the only entrance/exit of the nest cavity, therefore reflecting a female’s tendency for fear-induced defensive behaviour. Hence, our findings raise the possibility that the serotonergic system might be differentially involved in the expression of same-sex offensive aggression and fear-induced defensive behaviour in an avian species. Yet, this remains to be determined since the observed differential involvement of *SERT* might also be the result of differences in the heritability of our focal behavioural traits, which we were unfortunately unable to estimate due to the relatively low number of related females in our data. Nonetheless, repeatability estimates are useful in this regard as they enable the quantification of the upper limit to heritability^5^. Since cross-year repeatability is high for hissing behaviour (R = 0.64) but low for aggression (R = 0.19;^37^), it could hence have been more difficult to detect underlying loci for aggression than for hissing behaviour. Also, although variation in hissing behaviour has been linked to reproductive investment and success in our population^36,60^, it remains to be investigated whether the here detected synonymous *SERT* polymorphism are subject to selection^54^.

## Conclusion

Our findings suggest that *SERT* genotype might play a potential role in the expression of hissing behaviour, but replicated studies are necessary to verify our results. Yet, the two here identified SNPs with small effect provide valuable starting points in continuing to reveal underlying biological pathways involved in the expression of hissing behaviour. In contrast, we found no support for associations between *SERT* genotype and female same-sex aggression, even when assessing potential age-dependent effects. Whether age-dependent associations occur between aggression and other genes, including the role of epigenetic mechanisms on age-dependent behavioural trait expression, remains to be evaluated. In general, since genotype-by-age interactions are expected to be common for behaviour^15^, there is a clear scope for future molecular genetic studies to consider genotype-by-age interactions for behavioural trait (co)variation. With our study, we hope to stimulate research in this direction as this will vastly improve the proximate and ultimate understanding of the development of personality variation in natural populations.

## Supplementary Information

Supplementary Tables.

## Data Availability

The datasets generated during and/or analysed during the current study are available from the corresponding author on reasonable request.
