# Supplementary material for: The serotonin transporter gene and female personality variation in a free-living passerine
Source: Sci Rep. 2021 Apr 21;11:8577. doi: 10.1038/s41598-021-88225-4 (PMC8060275; doi:10.1038/s41598-021-88225-4)
Supplement: Supplementary file 1 — Supplementary Tables. [file 41598_2021_88225_MOESM1_ESM.docx]

**Supplementary information:**

**The serotonin transporter gene and female personality variation in a free-living passerine**

Bert Thys, Andrea S. Grunst, Nicky Staes, Rianne Pinxten, Marcel Eens, Melissa L. Grunst

Page

CONTENT 1

Table S1 Sequences and product length for *SERT* primers 2

Table S2 *SERT* single nucleotide polymorphisms 3

Table S3 Linkage disequilibrium statistics 5

Table S4 PCA loadings for aggression parameters 6

Table S5 Additive and overdominant effect models for female aggression 7

Table S6 Additive and overdominant effect models for hissing behaviour 8

**Table S1:** Sequences and product length (PL) for the primers used to amplify the 13 exons of the great tit *SERT* gene.

| **Region** | **Forward primer** | **Reverse primer** | **PL (bp)** |
| --- | --- | --- | --- |
| Exon 1 | AAAGGTGTGGGAAGACAAA | CTGTCATTATCCTATCCC | 661 |
| Exon 2 | GGTGTATCTGGAGTTTTAGGAAAG | ATGAGAAATCCCACAGTAATTCAG | 134 |
| Exon 3 | CAAGATTTGAAGTGATTTGAAGTGA | TATCTCTGAAGTCACAAGAAATGC | 219 |
| Exon 4 | CTTTATTGCTTGGATAGGAGTAGC | GAAATGTCGTGATTTTGAAAGCTG | 138 |
| Exon 5 | AAGCTAAATTGAGGGTGGACT | GCTCTCTGGGCAGGAACAAC | 134 |
| Exon 6 | ATTTTGAGGTAGACAACACAGGA | CTAAGATTGTGTCAGAAGTGCAA | 103 |
| Exon 7 | CAATCTGGCTTGTAATCATGGTA | GATACAGCCAGCATTCAATCC | 127 |
| Exon 8 | CAGCATGACAGTGACAAATCTC | GGACACTTTTACACAATACAGCT | 112 |
| Exon 9 | TCATTTTGCAACATATTTCCTAGTG | CACTAAATCCCCACCCTAAAGA | 131 |
| Exon 10 | CTCCTGTTACTTTTAGATGCCTG | CTGTAAAATCCCCTTTGTTCACT | 96 |
| Exon 11 | GTCCTTAAAACTGTGCTTTAGTAAC | GCATCTAAAAGTAACAGGAGGTATA | 100 |
| Exon 12 | AAAGCGTGTGCCATCAAA | CCTAAGAGTCAAACAAGCAAC | 221 |
| Exon 13 | TGCTCAGTTCAGCCTGTTGGA | CAGAGGCCTGAAACGCTCCT | 74 |

**Table S2**: Twenty-seven single nucleotide polymorphisms (SNPs) detected in a population of great tits in Antwerp (Fort 6/Campus Drie Eiken), Belgium. For each SNP we give the location, total sample size (N), major/minor alleles (M/m) with sample sizes per genotype and minor allele frequency (%m). SNPs with minor allele frequency >10% are depicted in bold (see Table 1).

| **Locus** | **Location** | **N** | **M/m** | **mm** | **Mm** | **MM** | **%m** |
| --- | --- | --- | --- | --- | --- | --- | --- |
| **SNP106** | exon 1 | 286 | G/A | 12 | 61 | 213 | 14.86 |
| SNP153 | exon 1 | 289 | A/C | 0 | 3 | 286 | 0.52 |
| **SNP163** | exon 1 | 286 | G/A | 5 | 57 | 224 | 11.71 |
| **SNP187** | exon 1 | 289 | C/T | 11 | 98 | 180 | 20.76 |
| **SNP226** | exon 1 | 289 | T/A | 32 | 114 | 143 | 30.80 |
| SNP252 | exon 1 | 292 | G/A | 0 | 1 | 291 | 0.17 |
| SNP313 | exon 1 | 292 | G/A | 0 | 3 | 289 | 0.51 |
| SNP490 | exon 1 | 292 | G/A | 2 | 13 | 277 | 2.91 |
| SNP32 | exon 2 | 301 | C/A | 0 | 26 | 275 | 4.32 |
| SNP35 | exon 2 | 301 | C/T | 20 | 4 | 277 | 7.31 |
| SNP35 | exon 3 | 303 | C/T | 0 | 4 | 299 | 0.66 |
| SNP100 | exon 3 | 303 | C/T | 0 | 39 | 264 | 6.44 |
| **SNP101** | exon 3 | 303 | A/G | 18 | 30 | 255 | 10.89 |
| **SNP125** | exon 3 | 301 | A/G | 17 | 45 | 239 | 13.12 |
| SNP170 | exon 3 | 303 | C/G | 1 | 55 | 247 | 9.41 |
| **SNP187** | exon 3 | 303 | A/T | 17 | 50 | 236 | 13.86 |
| SNP51 | exon 5 | 277 | C/T | 0 | 30 | 247 | 5.42 |
| **SNP36** | exon 6 | 286 | T/C | 72 | 140 | 74 | 49.65 |
| SNP45 | exon 9 | 289 | C/T | 0 | 2 | 287 | 0.35 |
| SNP48 | exon 9 | 290 | G/A | 0 | 29 | 261 | 5.00 |
| **SNP51** | exon 9 | 284 | C/T | 15 | 88 | 181 | 20.77 |
| SNP52 | exon 9 | 290 | G/A | 0 | 5 | 285 | 0.86 |
| SNP60 | exon 9 | 290 | C/A | 0 | 1 | 289 | 0.17 |
| **SNP84** | exon 9 | 289 | T/C | 58 | 124 | 107 | 41.52 |
| **SNP144** | exon 12 | 303 | C/T | 6 | 57 | 240 | 11.39 |
| SNP145 | exon 12 | 303 | G/A | 0 | 7 | 296 | 1.16 |
| **SNP66** | exon 13 | 302 | C/T | 26 | 115 | 161 | 27.65 |

**Table S3**: Linkage disequilibrium statistics (D’; Pearson’s r) between 8 SNPs in *SERT*. Different significance levels for linkage disequilibrium, after correcting for false discovery rate (FDR), are indicated in italics (P.adjust > 0.05) and bold (P.adjust > 0.01).

|  |  | exon 1 | | exon 6 | exon 9 | | exon 12 | exon 13 |
| --- | --- | --- | --- | --- | --- | --- | --- | --- |
|  |  | SNP187 | SNP226 | SNP36 | SNP51 | SNP84 | SNP144 | SNP66 |
| exon 1 | SNP163 | **0.998; -0.186** | **0.998; -0.242** | 0.226; 0.083 | *0.680; -0.127* | *0.354; -0.109* | 0.005; -0.001 | *0.717; 0.162* |
|  | SNP187 |  | **0.573; 0.440** | 0.150; 0.078 | 0.064; -0.017 | 0.005; 0.003 | *0.649; -0.119* | 0.034; 0.028 |
|  | SNP226 |  |  | 0.080; 0.054 | 0.118; -0.040 | 0.031; 0.025 | *0.197; 0.106* | 0.052; 0.048 |
| exon 6 | SNP36 |  |  |  | 0.127; -0.065 | 0.104; -0.086 | 0.148; -0.053 | 0.104; 0.065 |
| exon 9 | SNP51 |  |  |  |  | **0.776; 0.473** | *0.281; 0.197* | **0.174; 0.144** |
|  | SNP84 |  |  |  |  |  | 0.204; 0.087 | **0.202; 0.149** |
| exon 12 | SNP144 |  |  |  |  |  |  | 0.131; 0.076 |

**Table S4:** PCA loadings for aggression parameters scored during territorial intrusion in female great tits (N_females_ = 290; N_observations_ = 686) on the first two principal components. Approach distance was multiplied by -1 prior to analysis.

|  | PC1 | PC2 |  |
| --- | --- | --- | --- |
| Eigen value | 1.40 | 0.95 |  |
| Proportion total variance | 0.49 | 0.23 |  |
| No. of alarm calls | -0.317 | -0.700 |  |
| Approach distance | 0.332 | 0.537 |  |
| Time on decoy | 0.646 | -0.235 |  |
| No. attacks | 0.610 | -0.407 |  |

**Table S5**: Output of (A) additive and (B) overdominant effect models for female-female aggression. Significant effects are in bold.

|  | **exon 1** | | | **exon 6** | **exon 9** | | **exon 12** | **exon 13** |
| --- | --- | --- | --- | --- | --- | --- | --- | --- |
|  | **SNP163** | **SNP187** | **SNP226** | **SNP36** | **SNP51** | **SNP84** | **SNP144** | **SNP66** |
| **(A)** |  |  |  |  |  |  |  |  |
| Fixed | β (SE) | β (SE) | β (SE) | β (SE) | β (SE) | β (SE) | β (SE) | β (SE) |
| Intercept | **0.22 (0.10)** | **0.25 (0.10)** | **0.31 (0.11)** | 0.22 (0.13) | 0.18 (0.11) | **0.30 (0.12)** | **0.21 (0.10)** | **0.28 (0.11)** |
| SNP | 0.05 (0.10) | -0.09 (0.08) | -0.13 (0.08) | 0.02 (0.09) | 0.18 (0.11) | -0.05 (0.08) | 0.12 (0.10) | -0.07 (0.09) |
| Year 2017 | 0.13 (0.12) | 0.14 (0.12) | 0.12 (0.12) | 0.12 (0.12) | 0.15 (0.12) | 0.12 (0.12) | 0.13 (0.12) | 0.12 (0.12) |
| Year 2018 | **0.25 (0.12)** | **0.25 (0.12)** | **0.25 (0.12)** | 0.23 (0.12) | 0.20 (0.12) | 0.23 (0.12) | 0.21 (0.12) | 0.22 (0.12) |
| Year 2019 | -0.17 (0.12) | -0.19 (0.12) | -0.21 (0.12) | -0.11 (0.12) | -0.12 (0.12) | -0.11 (0.12) | -0.16 (0.12) | -0.19 (0.12) |
| Age 2 | **-0.47 (0.10)** | **-0.45 (0.10)** | **-0.52 (0.13)** | **-0.41 (0.16)** | **-0.47 (0.12)** | **-0.54 (0.15)** | **-0.46 (0.10)** | **-0.56 (0.13)** |
| Age 3+ | **-0.61 (0.11)** | **-0.60 (0.10)** | **-0.58 (0.15)** | **-0.81 (0.18)** | **-0.53 (0.13)** | **-0.59 (0.16)** | **-0.63 (0.11)** | **-0.64 (0.14)** |
| SNP x age 2 | - | - | 0.06 (0.15) | -0.06 (0.13) | -0.08 (0.17) | 0.05 (0.14) | - | 0.17 (0.14) |
| SNP x age 3+ | - | - | -0.04 (0.16) | 0.17 (0.15) | -0.30 (0.19) | -0.07 (0.15) | - | 0.05 (0.16) |
| Random | σ² (SE) | σ² (SE) | σ² (SE) | σ² (SE) | σ² (SE) | σ² (SE) | σ² (SE) | σ² (SE) |
| ID | 0.16 (0.02) | 0.16 (0.02) | 0.16 (0.02) | 0.17 (0.02) | 0.18 (0.02) | 0.17 (0.02) | 0.17 (0.02) | 0.17 (0.02) |
| ID_Year | 0.20 (0.03) | 0.21 (0.03) | 0.21 (0.03) | 0.19 (0.02) | 0.19 (0.03) | 0.19 (0.02) | 0.19 (0.03) | 0.18 (0.03) |
| Residual | 0.51 (0.03) | 0.51 (0.03) | 0.51 (0.03) | 0.56 (0.03) | 0.57 (0.03) | 0.57 (0.03) | 0.56 (0.03) | 0.56 (0.03) |
| **(B)** |  |  |  |  |  |  |  |  |
| Fixed | β (SE) | β (SE) | β (SE) | β (SE) | β (SE) | β (SE) | β (SE) | β (SE) |
| Intercept | **0.24 (0.10)** | **0.24 (0.10)** | **0.26 (0.11)** | **0.28 (0.12)** | **0.21 (0.11)** | 0.21 (0.11) | **0.20 (0.10)** | **0.25 (0.11)** |
| SNP | -0.05 (0.12) | -0.05 (0.10) | -0.10 (0.12) | -0.07 (0.12) | 0.15 (0.13) | 0.10 (0.12) | 0.17 (0.12) | -0.02 (0.12) |
| Year 2017 | 0.13 (0.12) | 0.14 (0.12) | 0.14 (0.12) | 0.10 (0.12) | 0.12 (0.12) | 0.11 (0.12) | 0.13 (0.12) | 0.11 (0.12) |
| Year 2018 | **0.25 (0.12)** | **0.25 (0.12)** | **0.24 (0.12)** | 0.22 (0.12) | 0.19 (0.12) | 0.22 (0.12) | 0.22 (0.12) | 0.21 (0.12) |
| Year 2019 | -0.17 (0.12) | -0.19 (0.12) | -0.21 (0.12) | -0.14 (0.12) | -0.12 (0.12) | -0.12 (0.12) | -0.16 (0.12) | -0.19 (0.12) |
| Age 2 | **-0.47 (0.10)** | **-0.45 (0.10)** | **-0.39 (0.12)** | **-0.57 (0.13)** | **-0.39 (0.12)** | **-0.54 (0.13)** | **-0.47 (0.10)** | **-0.46 (0.12)** |
| Age 3+ | **-0.60 (0.11)** | **-0.60 (0.10)** | **-0.62 (0.14)** | **-0.58 (0.15)** | **-0.60 (0.13)** | **-0.58 (0.15)** | **-0.63 (0.11)** | **-0.64 (0.13)** |
| SNP x age 2 | - | - | -0.14 (0.20) | 0.23 (0.20) | -0.35 (0.22) | 0.11 (0.20) | - | 0.01 (0.20) |
| SNP x age 3+ | - | - | 0.06 (0.21) | -0.13 (0.22) | -0.18 (0.24) | -0.16 (0.22) | - | 0.07 (0.22) |
| Random | σ² (SE) | σ² (SE) | σ² (SE) | σ² (SE) | σ² (SE) | σ² (SE) | σ² (SE) | σ² (SE) |
| ID | 0.16 (0.02) | 0.16 (0.02) | 0.16 (0.02) | 0.17 (0.02) | 0.19 (0.03) | 0.19 (0.02) | 0.17 (0.02) | 0.17 (0.02) |
| ID_Year | 0.20 (0.03) | 0.21 (0.03) | 0.20 (0.03) | 0.19 (0.03) | 0.18 (0.02) | 0.18 (0.02) | 0.19 (0.03) | 0.18 (0.02) |
| Residual | 0.51 (0.03) | 0.51 (0.03) | 0.51 (0.03) | 0.56 (0.03) | 0.57 (0.03) | 0.57 (0.03) | 0.56 (0.03) | 0.56 (0.03) |

**Table S6**: Output of (A) additive and (B) overdominant effect models for hissing behaviour. Significant effects are in bold. Effects that were significant at α = 0.05, but not after correcting for multiple testing are in italics.

|  | exon 1 | | | exon 6 | exon 9 | | exon 12 | exon 13 |
| --- | --- | --- | --- | --- | --- | --- | --- | --- |
|  | SNP163 | SNP187 | SNP226 | SNP36 | SNP51 | SNP84 | SNP144 | SNP66 |
| **(A)** |  |  |  |  |  |  |  |  |
| Fixed | β (SE) | β (SE) | β (SE) | β (SE) | β (SE) | β (SE) | β (SE) | β (SE) |
| Intercept | 0.19 (0.30) | 0.23 (0.31) | 0.23 (0.31) | 0.20 (0.31) | 0.31 (0.30) | 0.26 (0.31) | 0.25 (0.30) | 0.28 (0.30) |
| SNP | -0.06 (0.11) | 0.07 (0.10) | 0.04 (0.08) | 0.08 (0.07) | -0.01 (0.10) | 0.06 (0.07) | *-0.25 (0.11)* | 0.08 (0.08) |
| Lay date | **-0.12 (0.04)** | **-0.14 (0.04)** | **-0.14 (0.04)** | **-0.13 (0.04)** | **-0.15 (0.04)** | **-0.16 (0.04)** | **-0.13 (0.04)** | **-0.13 (0.04)** |
| Year 2017 | **0.42 (0.09)** | **0.42 (0.09)** | **0.42 (0.09)** | **0.41 (0.09)** | **0.44 (0.09)** | **0.40 (0.09)** | **0.42 (0.09)** | **0.40 (0.09)** |
| Year 2018 | **0.40 (0.10)** | **0.40 (0.10)** | **0.40 (0.10)** | **0.38 (0.10)** | **0.36 (0.09)** | **0.37 (0.10)** | **0.38 (0.09)** | **0.39 (0.10)** |
| Year 2019 | **0.53 (0.11)** | **0.53 (0.11)** | **0.53 (0.11)** | **0.54 (0.11)** | **0.45 (0.10)** | **0.48 (0.11)** | **0.50 (0.10)** | **0.51 (0.10)** |
| Mean clutch | -0.06 (0.04) | **-0.07 (0.04)** | **-0.07 (0.04)** | **-0.08 (0.04)** | **-0.08 (0.04)** | **-0.08 (0.04)** | -0.06 (0.04) | **-0.08 (0.03)** |
| Clutch dev | -0.03 (0.04) | -0.03 (0.04) | -0.03 (0.04) | -0.03 (0.04) | -0.04 (0.04) | -0.03 (0.05) | -0.04 (0.04) | -0.03 (0.04) |
| Random | σ² (SE) | σ² (SE) | σ² (SE) | σ² (SE) | σ² (SE) | σ² (SE) | σ² (SE) | σ² (SE) |
| ID | 0.63 (0.05) | 0.65 (0.05) | 0.65 (0.05) | 0.64 (0.05) | 0.68 (0.05) | 0.63 (0.05) | 0.68 (0.05) | 0.66 (0.05) |
| ID_Year | 0.12 (0.02) | 0.12 (0.02) | 0.12 (0.02) | 0.12 (0.02) | 0.10 (0.02) | 0.12 (0.02) | 0.10 (0.02) | 0.12 (0.02) |
| Residual | 0.19 (0.02) | 0.20 (0.02) | 0.20 (0.02) | 0.19 (0.02) | 0.19 (0.02) | 0.19 (0.02) | 0.19 (0.02) | 0.19 (0.02) |
| **(B)** |  |  |  |  |  |  |  |  |
| Fixed | β (SE) | β (SE) | β (SE) | β (SE) | β (SE) | β (SE) | β (SE) | β (SE) |
| Intercept | 0.18 (0.30) | 0.26 (0.31) | 0.23 (0.31) | 0.22 (0.31) | 0.35 (0.30) | 0.29 (0.30) | 0.23 (0.30) | 0.23 (0.30) |
| SNP | -0.01 (0.13) | 0.00 (0.11) | 0.07 (0.11) | 0.09 (0.10) | -0.14 (0.12) | 0.05 (0.10) | -0.22 (0.14) | *0.28 (0.10)* |
| Lay date | **-0.13 (0.04)** | **-0.14 (0.04)** | **-0.14 (0.04)** | **-0.12 (0.04)** | **-0.15 (0.04)** | **-0.16 (0.04)** | **-0.13 (0.04)** | **-0.13 (0.04)** |
| Year 2017 | **0.42 (0.09)** | **0.42 (0.09)** | **0.43 (0.09)** | **0.40 (0.09)** | **0.44 (0.09)** | **0.40 (0.09)** | **0.42 (0.09)** | **0.40 (0.09)** |
| Year 2018 | **0.40 (0.10)** | **0.40 (0.10)** | **0.40 (0.10)** | **0.37 (0.10)** | **0.37 (0.09)** | **0.37 (0.10)** | **0.37 (0.09)** | **0.39 (0.10)** |
| Year 2019 | **0.53 (0.11)** | **0.53 (0.11)** | **0.53 (0.11)** | **0.54 (0.11)** | **0.45 (0.10)** | **0.48 (0.11)** | **0.49 (0.10)** | **0.51 (0.10)** |
| Mean clutch | -0.06 (0.04) | **-0.07 (0.04)** | **-0.07 (0.04)** | **-0.08 (0.04)** | **-0.08 (0.04)** | **-0.08 (0.04)** | -0.06 (0.04) | **-0.08 (0.03)** |
| Clutch dev | -0.03 (0.04) | -0.03 (0.04) | -0.03 (0.04) | -0.03 (0.04) | -0.05 (0.04) | -0.03 (0.05) | -0.04 (0.04) | -0.03 (0.04) |
| Random | σ² (SE) | σ² (SE) | σ² (SE) | σ² (SE) | σ² (SE) | σ² (SE) | σ² (SE) | σ² (SE) |
| ID | 0.63 (0.05) | 0.65 (0.05) | 0.65 (0.05) | 0.64 (0.05) | 0.67 (0.05) | 0.63 (0.05) | 0.69 (0.05) | 0.64 (0.05) |
| ID_Year | 0.12 (0.02) | 0.15 (0.02) | 0.12 (0.02) | 0.12 (0.02) | 0.10 (0.02) | 0.13 (0.02) | 0.10 (0.02) | 0.12 (0.02) |
| Residual | 0.19 (0.02) | 0.20 (0.02) | 0.20 (0.02) | 0.19 (0.02) | 0.19 (0.02) | 0.19 (0.02) | 0.19 (0.02) | 0.19 (0.02) |
